# Effectiveness of a Bullying Intervention (Be-Prox) in Norwegian Early Childhood and Education Care Centers: Protocol for a Cluster Randomized Controlled Trial

**DOI:** 10.2196/60626

**Published:** 2024-10-24

**Authors:** Ingrid Kvestad, Frode Adolfsen, Renira Corinne Angeles, Oda Lekve Brandseth, Kyrre Breivik, Janne Grete Evertsen, Irene Kvåle Foer, Morten Haaland, Birgit Millerjord Homola, Gro Elisabeth Hoseth, Josefine Jonsson, Egil Kjerstad, Henriette Kyrrestad, Monica Martinussen, Annelene Moberg, Karianne Moberg, Anita Skogstrand, Line Remme Solberg, Merete Aasheim

**Affiliations:** 1 NORCE Norwegian Research Center Bergen Norway; 2 Regional Centre for Child and Youth Mental Health and Child Welfare, North UiT The Arctic University of Norway Tromsø Norway; 3 Bjørnafjorden municipality Bjørnafjorden Norway; 4 Narvik municipality Narvik Norway

**Keywords:** peer bullying in early childhood education and care, The Bernese Program, cluster randomized controlled trial, bullying, child, preschool, program evaluation

## Abstract

**Background:**

A new and growing body of research has studied bullying among children in early childhood education and care centers (ECECs). The Bernese Program (Be-Prox) is designed to systematically prevent and handle bullying between children in Swiss ECECs. However, the effectiveness of the Be-Prox intervention has not yet been explored in a Norwegian ECEC setting.

**Objective:**

This study aims to evaluate the effectiveness of Be-Prox in preventing and handling bullying among peers in Norwegian ECECs.

**Methods:**

ECECs from 2 Norwegian municipalities were invited to participate in a cluster randomized controlled trial (RCT) to evaluate the effectiveness of the Be-Prox intervention on peer bullying in Norwegian ECECs. After baseline measures were taken, project ECECs were randomized to either an intervention or a control arm. The Be-Prox intervention was introduced to ECECs in the intervention arm through 6 modules over a 9-month period immediately after the randomization. ECECs in the control arm participated in the data collection and were offered the Be-Prox intervention the following year. The primary outcome of the effect evaluation is the mean sum of negative behavior between peers after the Be-Prox training is completed in the intervention arm. Secondary outcomes include child bystander behavior, teacher self-efficacy, and ECEC’s authoritative climate. An extensive implementation and process evaluation, as well as cost-effectiveness analyses, will be conducted alongside the RCT.

**Results:**

Baseline data collection was conducted in September 2023, and the postintervention data collection started in May 2024. At baseline, we collected data on 708 children and 413 personnel from 38 project ECECs in the 2 Norwegian municipalities. The results from the study will be available in late 2024 at the earliest.

**Conclusions:**

The proposed project includes a comprehensive evaluation of the effectiveness of Be-Prox in Norwegian ECECs directly targeting the prevention and handling of bullying, including implementation and cost-effectiveness evaluations. The results from the project have the potential to fill in identified knowledge gaps in the understanding of negative behavior and bullying between peers in ECECs, and how these may be prevented. If proven efficient, our ambition is to offer Be-Prox to Norwegian ECECs as an evidence-based practice to prevent and handle bullying among preschool children.

**Trial Registration:**

ClinicalTrials.gov NCT06040437; https://clinicaltrials.gov/study/NCT06040437

**International Registered Report Identifier (IRRID):**

DERR1-10.2196/60626

## Introduction

According to the Norwegian Kindergarten Act (sections 41-43), Norwegian early childhood education and care centers (ECECs) should work systematically to prevent bullying and social exclusion, including adopting a zero-tolerance for violations, such as exclusion, bullying, violence, discrimination, and harassments [1].

Intentionally exposing other children to negative behavior is regarded as aggressive behavior [2,3]. Children with difficulties in regulating and understanding emotions are at greater risk of displaying aggressive behavior [4,5], as they may often misunderstand the emotions of others and are more inclined to interpret others’ intentions as aggressive [6]. Aggression can also be more planned and proactive, which is instrumental in reaching a goal, such as dominating others [7]. Children struggling to regulate emotions and behavior require developmental support from adults to function in interactions with their peers [8,9]. Preventing trajectories of aggressive behavior early gives a better prognosis later in life [10], and social and cognitive skills that children acquire in early childhood lay the foundation for later peer interactions and relationships [3,11]. Efforts to reduce the number of children exposing others or being exposed by others to aggression, as early as preschool years, are, in other words, important measures to promote social participation that may prevent social exclusion later in life.

A systematic review of universal social and emotional learning interventions for children in ECECs through 12th grade showed that children who participated in these interventions experienced improved academic achievement, school climate, school functioning, social and emotional skills, attitudes, and prosocial and civic behaviors, and reduced internalizing and externalizing problems [12]. The findings specifically suggest the value of teaching emotional skills before social skills, contributing to the strongest effects of the social and emotional learning programs [13,14]. In Norway, Fossum et al [15] used a randomized controlled between-group design to identify the preventive effects of the Incredible Years Teacher Classroom Management program on social competence and behavior problems of preschoolers. These findings suggested an increase in social competence and a reduction in aggression, internalizing, and attention problems among children. In addition, significant improvements in social competence were observed in a subsample of children who exhibited baseline aggressive behavior scores at or above the 90th percentile [15].

Aggressive behavior does not constitute bullying in itself. The term peer bullying is conventionally defined as aggressive behavior that occurs repeatedly over time and where there is a power imbalance between the child exposing others and the child being exposed [16]. While bullying is well-described in school-age children, a new and growing body of research also describes bullying among children in ECECs. Estimating the prevalence of bullying among children in ECECs based on previous studies is challenging, however, due to differences in how bullying is defined and operationalized in this age group, differences in informants, instruments used, and the modes of data collection [17]. Moreover, the concept of bullying among preschool children in ECECs is debated [2,18,19]. Some argue that children in this age group (ie, under 6 years) seem to aggress toward their peers in a rather indiscriminative way, and do not repeatedly target peers who have less power than themselves, which generally is considered an important criterion to discriminate bullying from aggression [3]. Hence, terms such as “unjustified aggression” [2] and “peer victimization” [20] have been suggested instead of bullying in this age group. Through results from their studies, Alsaker and Valkanover [21] argue that bullying also exists among children in kindergartens. In the Pathways to Victimization study, they used the concepts of teacher-reported physical, verbal, relational, and object-related negative acts and categorized children as victims, bullies, bully victims, and noninvolved [21].

Studies specifically targeting bullying between preschool peers are few and often limited by a low sample size [9]. In the Pathways to Victimization study, 6% of the children were found to be victims of bullying (ie, exposed to negative acts by other children at least once a week over a 3-month period), 11% were bullies (ie, exposing others to negative acts), 10% were bully victims (ie, both exposed and exposing others), and 46.5% were not involved [21,22]. Two Finnish studies found that 12.6% of children aged 3-6 years in ECECs were involved in bullying [23] and almost 30% of 4-year-olds [11], as reported by teachers and parents, respectively. From a Norwegian context, a scoping review found the prevalence in Norwegian ECECs varied from 6% to 20% in various studies [17]. A recent study, including approximately 900 children aged 1-5 years from Norwegian ECECs, used the concepts of physical, verbal, relational, and object-related negative acts to study negative behavior between peers and found that, across age, almost half of the children were involved in negative behaviors between peers, either as a victim, perpetrator, or both, 2-3 times a month or more often, based on teacher reports (unpublished data, ME Solberg et al, 2024). The prevalence varied markedly across age and the specific acts; however, with the prevalence of all types of negative acts increasing between those aged 1 and 2 years, after 3 years of age, the prevalence of physical negative acts declined, whereas the verbal and relational acts were attenuated.

There are limited evidence-based interventions described in the literature directly targeting bullying between peers in ECECs [3,17]. The Bernese Program (Be-Prox), designed to systematically prevent and handle bullying between children in Swiss ECECs, is one of the few interventions with supported evidence [24]. In Be-Prox, the aim is to increase understanding and skills among ECEC personnel through a 6-module training. An evaluation of the Be-Prox program in Swiss ECECs found a decrease in the number of children victimized after introducing the intervention, and that the risk of being victimized in the control ECECs was 1.5 times higher compared with ECECs where Be-Prox was introduced [25]. In a pilot study, Be-Prox was translated, adjusted, and evaluated for a Norwegian context, providing teaching materials and tools for ECECs in a Norwegian municipality. From this pilot, our experience is that Be-Prox is well accepted among Norwegian ECEC personnel and that the 6 modules were feasible in the effort to prevent and handle negative behavior and bullying in Norwegian ECECs, in accordance with the Norwegian Kindergarten Act [1]. However, whether the intervention leads to changes in the frequency of negative behavior and bullying in a Norwegian context is not known. To evaluate the effectiveness of the Be-Prox intervention to prevent and handle bullying in Norwegian ECEC, there is, therefore, a need for a sufficiently powered randomized controlled trial, preferably including several municipalities in different parts of Norway to increase the generalizability of the findings.

For successful implementation of an intervention in educational systems, such as ECECs, evidence should go beyond the solid empirical documentation of the effect of an intervention (evidence-based intervention) and incorporate the empirically based knowledge with the practitioner’s experience-based knowledge and the needs and wishes of the end users [26]. To be able to say with a reasonable degree of certainty that an intervention will be effectively used in ordinary practice, both positive results from good efficiency studies and evaluation of systems that ensure implementation quality when the intervention is used in ordinary practice are necessary. Therefore, the assessment of the implementation quality, fidelity, and usefulness of the program in question should be included in the study evaluation [27]. This is referred to as evidence-based practices, and the goal is to base decisions on useful interventions, measures, or programs on the best available scientific evidence aligned with the views and needs of the practitioners and end users [28]. More attention is also currently being directed to the economic evaluations of an intervention when implemented to, for instance, the educational system. These evaluations assess alternative program options in terms of cost and consequences. A core question is whether resources on interventions are optimally spent in terms of benefits gained, compared with the current practice [29]. Cost-effectiveness analysis compares the costs (monetary units, such as Norwegian Krone) and benefits (nonmonetary units, such as the prevalence of bullying) of the intervention with the standard practice [30].

The current project aims to evaluate the effectiveness of the evidence-based antibullying intervention, Be-Prox, in a cluster randomized controlled trial (RCT) in Norwegian ECECs with an overall aim to ensure a safe and sound ECEC environment for Norwegian children. We hypothesize that the Be-Prox intervention will be effective in preventing and handling bullying among peers in Norwegian ECECs and that it is possible to successfully implement the intervention in Norwegian municipalities.

We will reach the project aim through the project objectives in Norwegian ECECs, which are (1) to evaluate the effectiveness of the Be-Prox intervention to prevent and handle bullying among peers, (2) to examine implementation factors that promote or inhibit the effectiveness of the Be-Prox intervention, (3) to examine the cost-effectiveness of the Be-Prox intervention, and (4) to generate knowledge on how the Be-Prox intervention can be aligned and implemented in Norwegian municipalities.

## Methods

### Study Design and Setting

**Figure 1:**
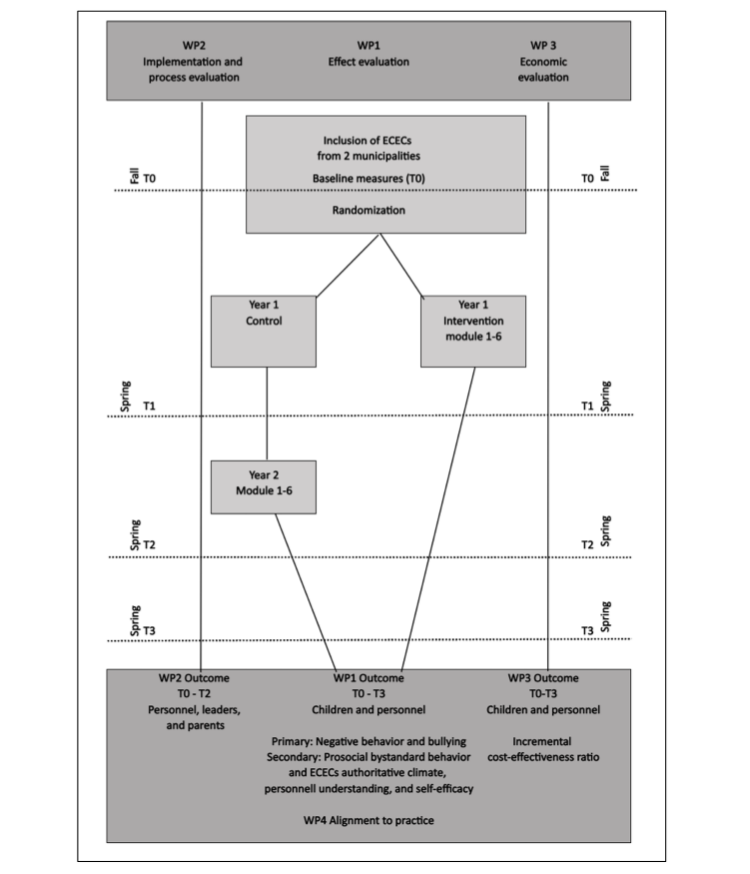
Overview of the Be-Prox study. Be-Prox: The Burmese Project; ECEC: early childhood education and care center.

The fundamental pillar of the project is the effect evaluation, using an RCT design, with longitudinal follow-ups in 2 Norwegian municipalities (Figure 1). The primary outcome for the effect evaluation will be measured after the training is complete in the intervention arm (T1). The implementation and process evaluation and economic evaluation will be conducted alongside this trial, benefitting from the stringently conducted RCT in a real-world setting, combining the advantages of minimizing selection bias and increasing the external validity of the findings [29].

The study is set in 2 mid-sized Norwegian municipalities, Bjørnafjorden and Narvik, with approximately 25,000 and 19,000 inhabitants and 24 and 28 ECECs, respectively [31]. Both public and private ECEC institutions were invited to participate in the study. The project adopts a pragmatic approach and includes all ECECs that were available and consented to participate.

### Participants and Recruitment

**Figure 2:**
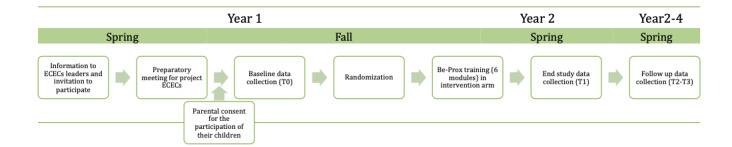
Study procedures in the Be-Prox study. Be-Prox: The Burmese Project; ECEC: early childhood education and care center.

All ECECs in the 2 municipalities received information about the project in meetings with the municipality and participants from the project group (Figure 2). While all public ECECs were included through the municipality, private ECECs consented to participation on their own after this initial meeting.

In ECECs consenting to participate (in the following referred to as project ECECs), parents of children that turned 3 years of age or older in project year 1 (ie, when ECECs in the intervention arm received the training) were asked to consent for the participation of their child to the study. Children were included if they belonged to a project ECEC, turned 3 years of age in project year 1 or were older, and if at least 1 parent consented to their participation. Children were excluded if they were younger than 3 years or if one parent specifically opposed the participation of their child.

All personnel in project ECECs that attended the Be-Prox training, as appointed by the head of the ECEC, were invited to participate in a general survey and evaluations of the Be-Prox training. Personnel who did not consent to participation were excluded.

All Be-Prox instructors in both municipalities were invited to fill in a self-evaluation following each Be-Prox training session. When training was completed in ECECs in the intervention arm (T1), all parents of children in the project ECECs were invited for an anonymous survey.

### Randomization and Allocation to Study Arms

Project ECECs were randomized to the intervention or control arm after baseline measures (T0) were completed about 1 month after the start of the ECEC year following the summer holidays (Figures 1 and 2). Randomization was stratified by municipality, the size of the ECEC (small or large), and public or private ECEC. Randomization was done by a statistician at UiT the Arctic University of Norway not otherwise involved in the project. A random number of 0 and 1 was generated using SPSS version 29.0 (IBM Corp) and assigned to each project ECEC.

### Intervention and Cointervention

#### Be-Prox Intervention

Drawing from an ecological approach focusing on the quality and context of the child’s environment [32], the Be-Prox intervention regards bullying among peers as a social phenomenon that requires both the presence of aggressive children and the passivity of other children and adults to develop into chronic patterns [25,33,34]. This intervention aims to lower the levels of bullying through a positive ECEC environment characterized by shared values and beliefs about behavior among children and ECEC personnel. Moreover, Be-Prox fosters an adult authoritative approach combining a high degree of support toward the children with a high degree of disciplinary structure. ECEC personnel, and in this regard, all adults working in ECECs independent of their profession, are, therefore, the main target group for the Be-Prox intervention [25]. The focus is on a 6-module training to increase understanding and skills in managing bullying among all personnel and encourage positive interactions between the children [34]. The goal is to prevent occurrences of negative behavior and bullying at an early stage, talk about bullying and victimization, strengthen prosocial resources in the group of children, and intervene when negative behavior and bullying occur [25].

The principles of Be-Prox, as described above, are introduced to ECEC personnel in 6 modules over a 9-month period [34] (Table 1). The Be-Prox training is conducted during personnel meetings throughout the ECEC year (usually modules 1 and 2 in 1 full day in early fall, then modules 3-6 in approximately 2.5-hour meetings, 2 in the fall and 2 during spring).

All participants in the training have access to the Be-Prox material and tools through the Be-Prox home page. Between the module training, there are assignments where the personnel try out and practice activities with the children. These assignments are discussed with colleagues at the start of the next module. After the training is completed, there is a final meeting in the ECEC leader team on the implementation of a quality system to ensure the sustainability of the program in the ECECs after training is completed.

**Table 1:** Be-Prox modules and program elements.

Modules	Title	Program elements
Module 1	Mobilize	To raise awareness of negative behavior and bullying among kindergarten peers
Module 2	Look at it	Uncover negative behavior and bullying among kindergarten peers
Module 3	Let’s talk about it	Discuss negative behavior and bullying with children, colleagues, and parents
Module 4	The contract	Involve children in making common rules of behavior
Module 5	Act and monitor	Kindergarten personnel are consistent in their follow-up of common rules
Module 6	Strengthening resources	Strengthening resources and prosocial bystander behavior
Program appendices	(1) Collaboration with parents. (2) Quality control systems to ensure the sustainability of the program	—^a^

^a^Not applicable.

#### Preparations

The leaders of all ECECs participated in a preparatory meeting (April and May in year 1) before the start of data collection, randomization, and training in the intervention arm (Figure 2). This meeting included brief information on the project, including data collection and randomization procedures, how to structure the training in the ECECs, and the importance of investing in the Be-Prox training in the first year for successful adoption of the program.

#### Instructor Training

The Be-Prox intervention was delivered to ECECs by local, trained instructors in pairs of 2. These local instructors were trained and supervised by senior advisors (AS, MH, MA, and JGE) throughout the intervention period.

Eligible candidates with competence at the bachelor’s or master’s level and relevant experience were identified within the municipalities to be trained as Be-Prox instructors. The instructor training was held over 6 days, with 3 days of introduction (August, year 1), 2 days of follow-up (January year 2), and a final digital 3-hour meeting summarizing and discussing the way forward (May and June year 2). The trained instructors (3 pairs in each municipality) are responsible for the Be-Prox training in the ECECs and receive digital supervision from senior advisors at the partner institutions, timed according to each module throughout the ECEC year.

#### Intervention Arm

In the intervention arm, a preparatory meeting with the ECEC leader team was conducted. After filling in the baseline measures (T0), ECECs randomized to the intervention arm continued with the full Be-Prox training over a period of about 9 months.

#### Cointervention Arm (Control)

In the cointervention arm, a preparatory meeting with the ECEC leader team was conducted and baseline measures (T0) were filled identical to the intervention arm. ECECs in the control arm will be offered the Be-Prox training in year 2.

### Data Collection

All data in the project are collected by questionnaires created with Nettskjema, a survey solution developed and hosted by the University of Oslo. Data are stored within the same system (TSD, Services for Sensitive Data, University of Oslo) designed for the storing and postprocessing of sensitive data in compliance with applicable regulations [35].

Data on child behavior are collected by 2 ECEC personnel who know the children well (as appointed by the ECEC leader). The questionnaire takes approximately 10 minutes to fill in for each child. These personnel received an introductory video on how to fill in the questionnaire. The personnel in all project ECEC will complete questionnaires on child behavior at baseline (T0) and following the end of the Be-Prox training in the intervention arm (T1). Data will also be collected after 1 (T2) and 2 (T3) years following the end of the Be-Prox training in the intervention arm (longitudinal follow-ups; Figures 1 and 2).

For personnel, we collect survey data at baseline (T0) and after completion of the training in the intervention arm (T1), and 1 (T2) and 2 (T3) years after the end of the Be-Prox training in the intervention arm (Figures 1 and 2). At each time point, questionnaires take approximately 30 minutes to fill in. During the intervention year, personnel will also fill in questions related to the training modules (fidelity) and the instructors will be requested to anonymously fill in fidelity checklists connected to each training module.

During the spring of year 2 (T1), we will invite all parents to fill in an anonymous user satisfaction survey.

### Outcomes

#### Effect Evaluation

##### Negative Behavior Between Peers

The frequency of negative behavior between peers will be rated by ECEC personnel using 4-item scales on physical, verbal, relational, and object-related negative acts, where 1 scale is on exposing peers to negative acts, and 1 scale is on being exposed to negative acts by peers [21]. These scales are adapted from the Pathways to Victimization study, have shown acceptable construct validity [36,37], and will have the following response categories in this study: “never or seldom” (0), “once per month” (1), “2-3 times per month” (2), “1 time per week” (3), and “more than once a week” (4) using the last month as the reference period.

In this study, the scales will be used as sum scores (ie, “exposing peers to negative behavior” and “being exposed to negative behavior by peers,” range 0-20), itemized by the different negative acts (ie, physical, verbal, relational, object-related, range 0-5) and as the frequency of children exposing peers and being exposed by peers to negative behavior more than 2-3 times per month. For each of the 8 negative acts, the questionnaire will also include an identical item assessing whether there was a power imbalance involved in the negative acts with the response categories no (0), yes (1), and yes and no (2).

The primary outcome of the effect evaluation are the mean sum of negative behavior (range 0-16), exposing peers and being exposed by peers, post intervention. This is also what forms the basis for the power calculations.

##### Child Bystander Behavior

Bystander behavior, that is, trying to help the exposed child, withdrawing from the situation, or joining in on the bullying will be assessed by a 12-item questionnaire adapted from the Pathways to Victimization study [38], with the response categories “never” (0), “seldom” (1), and “always” (2).

##### ECECs Personnels’ Self-Efficacy

Personnels’ self-efficacy and the probability that they will intervene will be assessed by adapted items from the Bullying Intervention Self-Efficacy Scale, developed and validated for a German school setting [39,40]. The original 5-item scale was translated to Norwegian for this study and wordings changed from school peers to ECEC peers. We also changed the response category from 4 to 7 points on the Likert scale, where high scores indicated high self-efficacy in handling bullying situations.

##### Authoritative Climate

Authoritative climate will be measured through an adapted version of a scale constructed and validated for Norwegian schoolteachers [41]. In this study, we have included 10 items where the personnel evaluate the degree of support and control in the ECEC environment on a 7-point Likert scale. From the original 8-item scale, adaption for the current ECEC environment included changing from using “I” to “Adults in our unit” and adding 2 items related to the ECEC context. In this scale, higher scores indicate a higher degree of authoritative ECEC climate.

#### Implementation and Process Evaluation

To identify factors that promote or inhibit the implementation of the Be-Prox program, the study will investigate issues regarding the personnel’s work environment, intervention fit, and organizational readiness for change [42,43]. Questions were rephrased to be more relevant to this study setting. For example, in addition to questions phrased in the first person (I), questions in the third person (them) were added. Wordings were also rephrased to address the current theme of “bullying” within this specific ECEC context. Questions related to employees’ perception of workload, work conflict and work-family conflict, and autonomy and leadership, as well as job satisfaction, were adjusted to this study and included [44]. Factor structure and psychometric properties of the Norwegian versions are supported in previous studies [45,46]. In addition, questions about employees’ intention to quit [47] and employees’ perceptions of burnout and engagement [48,49] will be examined. Questions are rated on a 7-point Likert scale ranging from 1 (strongly disagree) to 7 (strongly agree) or assessed from 1 (not at all) to 7 (to a very large extent).

Implementation quality, fidelity, and usefulness of the program will be assessed through checklists to Be-Prox instructors and module evaluations to personnel. Checklists and module evaluations were developed for this study based on Be-Prox materials and tools [34], and distribution was timed according to each training module, as well as a final evaluation after the last training module.

This study will also explore parents’ experiences of how the personnel addresses negative behavior and bullying, as well as parents’ confidence and ability to raise concerns about these issues through a user satisfaction survey. Questions are assessed on a 5-point scale ranging from 1 (to a very large extent) to 5 (to a very small extent) and are supported in previous Norwegian studies [50,51]. The survey will be distributed electronically to the parents after the program completion at the end of the first year (T1).

#### Economic Evaluation

The economic evaluation of Be-Prox will consist of a cost-effectiveness analysis from a societal and ECEC perspective. A cost-effectiveness analysis compares the costs and consequences of different programs, such as antibullying interventions, and estimates the value of the program [30]. The aim of the current study is to evaluate the cost-consequences for each gained unit effect of the Be-Prox intervention.

In the cost-effectiveness analysis, we will use the primary outcome as outlined above. The cost data for the cost-effectiveness analysis will include both direct and indirect intervention costs. Direct costs include material and labor costs for extra staff or extra labor hours used to implement the program incurred by the ECECs [30,52]. Indirect costs include labor costs that are not directly incurred by the kindergarten but compensate for the time used to implement the program that could have been otherwise spent on other core activities. These costs are relevant economic costs (“alternative costs”) and often constitute the largest part of the overall costs for similar intervention programs. The cost information will be collected from ECEC leaders at T3. Information on time resources and workload connected to the Be-Prox implementation will be collected as part of the fidelity measures.

#### Demographic Variables

Information on the child’s age and gender and parental country of birth will be collected from parents while asking for consent for child participation (before T0, Figure 2). Demographic information of the personnel, such as age, gender, nationality, educational background, current position in the ECEC, and years of experience from ECEC work will be collected in the survey at baseline (T0). Based on the leader’s report, organizational variables of the ECECs (eg, number of employees, children, and departments, type of organization and structure in the ECEC, and previous competence-enhancing courses and measures) will also be examined [45].

### Sample Size Estimations

A 2-level random intercept multilevel model will be used to detect group mean differences (intervention vs control) on posttest scores. Pretest scores aggregated at the cluster level will be included as covariances to increase power [53]. Based upon interclass correlations and cluster sizes from the pilot study, power calculation using the Optimal Design Software (Version 3.01) [54] shows that with a .05 level of significance, power of 0.80, expected ICC at kindergarten level at 0.12, a cluster size of 45 (children), and pretest cluster-level covariate explaining 30% of the posttest score, 39 kindergartens are needed to detect effect sizes equal to or larger than Cohen *d* 0.30m representing a medium effect [55]. Given the potential risk of dropout during the trial and potential contamination bias, we have decided to recruit at least 44 of the 52 available kindergartens.

### Statistical Analyses

For the effect evaluation (primary outcomes), 2-level random intercept multilevel models will be used to detect group mean differences in negative behavior (intervention versus control) on posttest scores. Pretest scores aggregated at the cluster level will be included as covariates to increase power [53]. Multilevel growth curve analyses will be conducted to explore whether potentially positive gains from the RCT are maintained at 1 and 2 years after the end of the intervention. Multilevel analyses will also be used in other analyses when data clustering must be accounted for. Missing data will be handled with multiple imputations and full information maximum likelihood [56].

Demographic information provides important descriptions of the samples and can facilitate measurements of potential effect modifications (eg, by age, gender, position, and educational background).

The implementation evaluation will examine whether implementation fidelity and quality (eg, mean evaluation of the Be-Prox training received by employees and mean evaluation of the training delivered by the Be-Prox instructors) predict the effectiveness of the Be-Prox intervention (ie, the difference in frequency of negative behaviors between baseline and T1). Multilevel models with ECEC as a cluster variable will be used. The experiences of the parents will also be analyzed with multilevel analyses (with ECEC as a cluster variable) to investigate which factors predict their satisfaction with how the personnel address negative behavior and bullying. The effect of the Be-Prox intervention on the employees’ attitudes and experiences of the work environment (eg total workload, attitude toward evidence-based programs, and autonomy) will be evaluated using 2-level random intercept multilevel models that will compare changes in the control and intervention groups through time (T0, T1, and T2).

The economic evaluation will calculate the cost-effectiveness of the intervention based on estimates from the incremental cost-effectiveness ratio (ICER). The ICER indicates the additional investments needed for the intervention to gain one extra unit of effect compared with control ECECs with no intervention, which can be interpreted as the monetary cost for 1 less child victim or offender [29]. When ICERs are estimated, we will use nonparametric bootstrapping with 4000 replications to estimate 95% CIs around cost differences and the uncertainty surrounding ICERs. To account for the clustering of data, bootstrap replications will be stratified by ECECs. Bootstrapped cost-effect pairs will be plotted on a cost-effectiveness plane and used to calculate cost-effectiveness acceptability curves. Cost-effectiveness acceptability curves display the probability that a treatment is cost-effective compared with control ECECs with no treatment. We will also carry out deterministic and probabilistic sensitivity analyses to establish the uncertainty of the cost-effectiveness results.

We will also use other statistical approaches, such as simple descriptive statistics, regression analyses, factor analysis, and structural equation models, to answer secondary research questions in the project.

### User Involvement

User knowledge is essential in the process of building knowledge for evidence-based practice [26]. In the current project, the perspectives of the end users will be incorporated through parent representatives and antibullying professionals in the reference group meeting yearly. The project will also involve ECEC practitioners in the planning of the study and interpretations of results and alignment to practice.

### Ethical Considerations

The study will be conducted in accordance with the ethical principles outlined in the Declaration of Helsinki. Due to the nature of the study, formal approval from an ethics committee was deemed not necessary. All participants were thoroughly informed about the study purpose and procedures and consent was collected through the Nettskjema. Participation in the study is voluntary and consent can be withdrawn at any time. Data will be handled according to recommendations from the Norwegian Data Protection Service (reference number 705199), with the public interest as the legal basis for processing personal data (ie, Art. 6 (1)(e) of the General Data Protection Regulation). All data will be collected and stored within systems designed for storing and postprocessing of sensitive data in compliance with applicable regulations (ie, Nettskjema and TSD). Only a restricted number of members from the project team will have access to raw data. The remaining project members will have access to anonymized data only. A Data Protection Impact Assessment has been conducted by the Norwegian Agency for Shared Services in Education and Research and the Data Protection Officer at NORCE, in collaboration with the principal investigator (IK) and co–principal investigator (MA). The processing of personal data was evaluated to be in line with the personal data protection regulations. The project is registered at ClinicalTrials.gov (NCT06040437).

## Results

The project secured funding in June 2022, started in January 2023, and received recommendations from the Norwegian Data Protection Service in July 2023. Baseline data collection was conducted in September 2023, and the postintervention data collection started in May 2024. From baseline, we have data from 708 children from 38 project ECECs in the 2 Norwegian municipalities and 413 of the personnel, constituting a response rate of 70% and 80% of all available participants, respectively. Results from the study will be available in late 2024 at the earliest and will be published in peer-reviewed international and national journals, as well as presented at relevant conferences.

## Discussion

### Principal Findings

The proposed project includes a comprehensive evaluation of the effectiveness of Be-Prox in Norwegian ECECs directly targeting the prevention and handling of bullying. The evaluation includes a stringent RCT design in a real-world municipality setting with implementation and cost-effectiveness evaluations.

Be-Prox is one of the few interventions described in the literature with scientific evidence for an effect on negative behavior and bullying between peers in kindergartens [9,24]. Through the existing project outputs, we will be able to measure whether such an effect also can be found in Norwegian ECECs. Including a wider range of outcome measures, we will be able to examine important mechanisms involved, such as the effect on negative behavior as such or on bullying as conventionally defined, the mediation by personnel factors, and the potential modification by age and gender. Hence, findings from the current project will represent an important contribution to the research field, both in providing scientific evidence as to whether Be-Prox is effective beyond Switzerland and on important mechanisms involved.

From the described project, we will also obtain comprehensive data on negative behavior and bullying between preschool peers in a large population-based sample from Norwegian ECECs. Data will be collected on multiple levels, including children, parents, personnel, and ECEC leaders. The concept of bullying in this age group is debated [2,3], and data from the current project can contribute to an increased understanding of the concept of bullying among ECEC children.

The importance of the project outputs is underlined by the additions to the Norwegian Kindergarten Act in 2021, introducing the right of all children to a safe and sound ECEC environment [1]. Although, increasingly also in Norway, there is a consensus that bullying exists among preschool children in ECECs, few interventions are available directly targeting bullying behavior in this setting [17]. The lack of evidence-based practice represents a challenge for Norwegian ECECs as well as the municipalities responsible for ECECs being run in accordance with applicable laws and regulations. Going beyond measuring the effect of Be-Prox to prevent and handle bullying; the current project also incorporates essential factors for successful implementation and to whether Be-Prox is a cost-effective alternative. In this regard, project outputs will provide practitioners and decision makers with information on whether the intervention fulfills its purpose and, in addition, inform on whether Be-Prox may be implemented in practice and at what cost. If effectiveness is proven with an effect size that is judged to be of practical significance, Be-Prox can be offered to ECECs and municipalities nationwide.

The negative consequences for children being exposed to bullying are well-described [57], and the available evidence suggests that this can also be generalized to ECEC children [58]. Research findings point to the importance of preventing trajectories of aggressive behavior early [10] and that social skills acquired in early childhood lay a foundation for later relationships [3]. Reducing the number of children experiencing or exposing others to negative acts or bullying may therefore be an important measure in a common effort to promote social participation and prevent social exclusion among the rising generation. In this regard, the project is not only answering to the United Nations Convention on the Rights of the Child stating the right for all children to be protected from all forms of physical and mental violence [59], but also to several of the Sustainable Development Goals (SDGs), including SDG 3 to ensure healthy lives and promote well-being at all ages and SDG 4 to ensure inclusive and equitable quality education to all [60].

### Strength and Limitations

To the best of our knowledge, this is one of few RCTs investigating the effect of an anti-bullying intervention in Norwegian ECECs. Strengths of the study include being a well-powered cluster randomized trial enrolling a large sample of children aged 3 to 5 years and adopting a pragmatic approach in a real-world setting increasing the external validity of our findings. The study setting of 2 Norwegian municipalities representing different geographical areas (western and northern Norway) should also be considered a study strength. The project adopts an interdisciplinary approach, with project group members from different disciplines (eg, psychology, kindergarten, pedagogy, and economics) and representing different work areas such as kindergarten authorities at the municipality level, scientists, and senior advisors. The project setup, with the evaluation of effect, implementation, process, and economics, facilitates a large data collection with the potential to not only increase the knowledge of peer bullying in Norwegian kindergartens but also to what extent the Be-Prox intervention may be implemented in Norwegian municipalities and to what cost.

Limitations to the study include the risk of loss of follow-ups of project ECECs, children, and personnel and loss of power to identify meaningful effects. Moreover, since control ECECs are offered the Be-Prox intervention in year 2 of the project, it is not possible to measure the long-term effects of the intervention using the RCT design. We will, however, through state-of-the-art statistical methods measure associations between the Be-Prox intervention and relevant outcomes in a longitudinal design.

### National and International Collaboration

The study is a collaboration between 2 regional centers in Norway working on mental health problems among children and adolescents: RKBU West/NORCE and RKBU North/UiT and 2 Norwegian municipalities: Bjørnafjorden and Narvik. The project is also conducted in close collaboration with the developer of the Be-Prox intervention, Professor Emerita Francoise Alsaker from the Department of Psychology, University of Bern, Switzerland.

### Conclusion

Results from the project have the potential to fill in identified knowledge gaps in the understanding of negative behavior and bullying between peers in ECECs and how these may be prevented. If proven efficient, our ambition is to offer Be-Prox to Norwegian ECECs as an evidence-based practice to prevent and handle bullying among preschool children, in accordance with the Norwegian Kindergarten Act [1]. In a broader context, the findings will have the potential to inform future strategies for combating bullying in kindergartens, and how, through a systematic measure, to promote social participation and avoid social exclusion later in life, thereby contributing to a safer and more inclusive learning environment for children in Norway.

## Appendix

Multimedia Appendix 1 Peer review report by the Forskingsrådet - Research Council of Norway.

## Abbreviations

Be-Prox: The Bernese Program
ECEC: early childhood education and care center
ICER: incremental cost-effectiveness ratio
RCT: randomized controlled trial
SDG: Sustainable Development Goals

## Data Availability

The data sets generated or analyzed during this study are available from the corresponding author upon reasonable request.
