# Supplementary material for: Effectiveness of a Bullying Intervention (Be-Prox) in Norwegian Early Childhood and Education Care Centers: Protocol for a Cluster Randomized Controlled Trial
Source: JMIR Res Protoc. 2024 Oct 24;13:e60626. doi: 10.2196/60626 (PMC11544344; doi:10.2196/60626)
Supplement: Multimedia Appendix 1 [file resprot_v13i1e60626_app1.pdf]

Prosjektvurdering – Karaktersammendrag og administrasjonens vurdering av relevans

Prosjektnummer: 336181
Prosjekttittel: Be-Prox. An effectiveness study of a bullying intervention in Norwegian kindergartens.

	Vurderingskriterium	Karakter
1	**Forskningskvalitet**	**6**
2	**Virkninger og effekter**	**6**
3	**Gjennomføring**	**6**
4	**Relevans for utlysningen**<br>**7** *Søknaden er innenfor de utlyste temaområdene, spesielt temaområde B. Søknaden oppfyller krav og forventninger til prosjektleder og samarbeidspartnere. Intervensjonen omfatter flere barnehager, kommuner og mobbeombud i fylkeskommuner. Søknaden har en klar samarbeidskonstellasjon og internasjonalt samarbeid. Søknaden oppfyller forventninger knyttet til kompetanseoppbygging og kunnskapsspredning på en svært god måte.*	**7**
	**Hovedkarakter***	**6**

Karakterskala: 7, 6, 5, 4, 3, 2, 1 (7 er best)

*) Hovedkarakter er et uttrykk for hvor godt prosjektet oppfyller intensjoner og formål for søknadstypen med utgangspunkt i bedømmelsen av de ulike kriteriene. Hovedkarakter 3 eller lavere forteller at prosjektet har en avgjørende svakhet i form av karakter 3, 2, 1 på minst ett av kriteriene.
"Relevans for utlysningen" betegnes "ikke vurdert" dersom søknaden ligger under administrativ terskel for en fullstendig behandling av søknadene.

Karakterene er en del av underlaget ved det besluttende organs behandling av søknaden.

Vurdering av søknad sendt til Norges forskningsråd

Søknad

Prosjektnummer	336181
Prosjekttittel	Be-Prox. An effectiveness study of a bullying intervention in Norwegian kindergartens.
Prosjektleder	Kvestad, Ingrid
Prosjektansvarlig	NORCE Samfunn/Helse VESTLAND
Søknadstype/variant	Kompetanse- og samarbeidsprosjekt / Samarbeid
Tema	Barnehage, skole og videregående opplæring
Program/Aktivitet	Samarbeidsprosjekt 2022
Saksbehandler	Tone Merete Holtan

Bekreftelse

Ved å levere dette skjema i utfylt stand, bekrefter jeg / vi følgende forhold (dette gjelder for den enkelte ekspert eller panelet):

- Jeg/vi er habil til å vurdere denne søknaden. Se Forskningsrådets bestemmelser om habilitet og tillit.	Ja
- Jeg/vi har lest og gjort meg/oss kjent med de kriteriene jeg/vi er bedt om å vurdere søknaden etter og retningslinjene for bruk av karakterskalaen. Karakterskalaen skal brukes for å reflektere absolutte verdier, og karakterer skal ikke settes relativt til andre søknader som panelet/fageksperten vurderer.	Ja
- Jeg/vi er kjent med og har akseptert bestemmelsene for vurdering av søknader for Norges Forskningsråd. Se Bestemmelser for eksperter / panel som skal vurdere søknader for Norges forskningsråd.	Ja
- Jeg/vi er kompetent(e) til å foreta denne vurderingen.	Ja

Karakteroversikt

Kriterier	Karakter
Forskningskvalitet | KSP	6
Virkninger og effekter | KSP	6
Gjennomføring | KSP	6

Kriterier

Forskningskvalitet | KSP

.

I hvilken grad prosjektet er ambisiøst, nyskapende og flytter forskningsfronten
• Vitenskapelig kreativitet og originalitet.
• I hvilken grad hypoteser og problemstillinger er nyskapende og dristige.
• I hvilken grad prosjektet har potensial for å frembringe ny kunnskap som flytter forskningsfronten, inkludert vesentlig utvikling/fornyelse av teori, metoder, eksperimenter eller empirisk kunnskap.

Kvaliteten på prosjektets FoU-aktiviteter
• Kvaliteten på problemstillingene, hypotesene og prosjektets mål, og i hvilken grad de er klart og tydelig beskrevet.
• I hvilken grad den teoretiske tilnærmingen, forskningsdesignet og metodevalget er troverdig og velegnet, og tverrfaglige innfallsvinkler er tilstrekkelig vurdert.
• I hvilken grad prosjektet tar hensyn til samfunnsansvar, etiske problemstillinger og kjønnsdimensjonen i forskningen på en tilfredsstillende måte.
• I hvilken grad prosjektet forholder seg til brukeres/interessenters kunnskap på en tilfredsstillende måte.

The proposed project is a randomized control trial of an existing bullying intervention (BeProx) in two municipalities, and the project is very diligently laid out. The project will be registered, which is an important asset.
The primary objective is to evaluate the effectiveness of Be-Prox to prevent and handle bullying among peers in Norwegian kindergartens. Secondary objectives are to examine implementation factors that promote or inhibit the effectiveness of Be-Prox , to describe the cost-effectiveness of the Be-Prox intervention, and to generate knowledge on how the Be-Prox intervention can be aligned and implemented in Norwegian kindergartens.
It is clear that every step of the project has been thoroughly thought out. The project will be preregistered, make use of cluster randomization (childcare centers), sample size is based on power calculations (they may want to consider recruiting all childcare centers in the two municipalities in order to be able to detect effect sizes lower than 0.30), mixed-effects models will take care of nesting issues, multiple imputation will take care of missing data. With the careful procedures, the study is likely to yield interpretable and important results. The group may want to reconsider whether they ought to give the control group staff the module 1 introduction to bullying issues. If staff in the control group raise awareness of bullying, this might influence the design's counter factual and reduce effect sizes. The project is obviously of high societal importance.
In summary, the project is well-written, ambitious and novel. The research design is thoroughly described and appropriate in terms of reaching the goal of the project.

Valgt karakter: 6 - Svært godt
Søknaden svarer svært godt på kriteriet. Den har svært få, uvesentlige, mangler.

Virkninger og effekter | KSP

.

Potensielle virkninger og effekter av den foreslåtte forskningen
• I hvilken grad prosjektets planlagte resultater kan bidra til å møte viktige vitenskapelige utfordringer, både nå og i fremtiden.
• I hvilken grad prosjektets planlagte resultater kan svare på viktige utfordringer i sektoren(e), både nå og i fremtiden.
• I hvilken grad kompetanseutviklingen og prosjektets planlagte resultater vil gi grunnlag for verdiskaping i norsk næringsliv og/eller offentlig sektor.
• I hvilken grad prosjektets planlagte resultater er relevante for FNs bærekraftsmål eller har potensiale for å bidra til å møte andre viktige samfunnsutfordringer, både nå og i fremtiden.
• I hvilken grad de potensielle virkningene og effektene er tydelig formulert og troverdige.

Kommunikasjon og utnyttelse
• Kvaliteten på, og omfanget av, kommunikasjons- og involveringsaktiviteter rettet mot relevante interessenter/brukere.
• I hvilken grad samarbeidspartnerne er involvert i arbeidet med å ta i bruk prosjektets resultater.

The result of the project in terms of the effect of the intervention and in terms of implementation factors for the effectiveness of the study will be important for improving the thriving of children in Norwegian childcare. The project also includes professional development of childcare staff. Thus, the study has the potential to contribute importantly to early child development, which is clearly an important societal contributions. The authors clearly articulated these assets of the study. The feedback loop between practitioners and the project group is valuable. It is, however, not quite clear at which time points feedback is given and received. The project outlines degree and nature of partner involvement, and partners such as municipalities and childcare centers will clearly benefit from the study; obviously mainly if the intervention proves successful. But even if unsuccessful, the answers to the questions pertaining to implementation factors will help bring the science on this topic further. In summary, the project is likely to impact both practice and research. The project has a clear plan for dissemination.

Valgt karakter: 6 - Svært godt
Søknaden svarer svært godt på kriteriet. Den har svært få, uvesentlige, mangler.

Gjennomføring | KSP

.

Kvalitet på prosjektleder og prosjektgruppe
• I hvilken grad prosjektleder har relevant kompetanse og erfaring og har vist evne til å utføre forskning av høy kvalitet (sett i forhold til hans/hennes karrierestadium).
• I hvilken grad prosjektdeltakerne utfyller hverandre, og i hvilken grad prosjektgruppen innehar den nødvendige kompetansen for å gjennomføre prosjektet på en effektiv måte.

Kvaliteten på prosjektets organisering og ledelse
• I hvilken grad prosjektets organisering er effektiv, inkludert i hvilken grad ressursene til de forskjellige arbeidspakkene er tilstrekkelig og i samsvar med prosjektets mål og leveranser.
• I hvilken grad oppgavene i prosjektet er fordelt på en måte som sikrer at alle prosjektdeltakere har en tydelig rolle og tilstrekkelig med ressurser til å fylle denne rollen.
• I hvilken grad ledelse og styring i prosjektet er organisert på en egnet måte.
• I hvilken grad samarbeidspartnerne bidrar i styringen og gjennomføringen av prosjektet.

The research team is quite experienced. The PI has experience with managing larger research project and RCTs. The PI has published extensively in high rank journals and seems very capable of managing the proposed study. The project group as a whole and the partners involved seem to possess the relevant skills and experience to ensure successful execution of the project. Each role is clearly described and the outlined project plan seems sound. A little caveat is that the project overall seems a little expensive for a randomized control trial of a preexisting intervention. These matters are inherently hard to evaluate, but perhaps two or three more municipalities could be added for more statistical power and a higher degree of generalizability of the results to other municipalities across Norway.

Valgt karakter: 6 - Svært godt
Søknaden svarer svært godt på kriteriet. Den har svært få, uvesentlige, mangler.

Sjekkpunkter

Kommentar til sjekkpunkter
